# A project to define standards for radiology staffing, education and training across the European Union

**DOI:** 10.1186/s13244-025-01924-8

**Published:** 2025-03-12

**Authors:** Adrian P. Brady, Boris Brkljačić, Jonathan McNulty, Christian Loewe, Mary Coffey, Dimitris Visvikis, Yavuz Anacak, Cristina Garibaldi, Monika Hierath, François Jamar, Núria Jornet, Pedro C. Lara, Michelle Leech, Graciano Paulo, Csilla Pesznyak, Irene Polycarpou, Roberto M. Sánchez, Martina Szucsich, Francis Zarb, on behalf of the European Society of Radiology, in Conjunction with ESTRO, EFRS, EFOMP and EANM

**Affiliations:** 1 Department of Radiology, University College Cork, Cork, T12 AK54 Ireland; 2 Department of Diagnostic and Interventional Radiology, University Hospital Dubrava, University of Zagreb School of Medicine, Zagreb, Croatia; 3 Radiography and Diagnostic Imaging Section, School of Medicine, University College Dublin, Dublin, Ireland; 4 Division of Cardiovascular and Interventional Radiology, Department of Biomedical Imaging and Image-Guided Therapy, Medical University of Vienna, Vienna, Austria; 5 Adjunct Associate Professor, Discipline of Radiation Therapy, School of Medicine, Trinity Centre for Health Sciences, St. James’ Hospital, Dublin 8, Ireland; 6 LaTIM, INSERM, UMR1101, University of Brest, Brest, France; 7 Department of Radiation Oncology, Ege University Faculty of Medicine, Izmir, Turkey; 8 Medical Physicist Deputy Director, Radiation Research Unit, IEO, European Institute of Oncology IRCCS, Milan, Italy; 9 European Society of Radiology, Vienna, Austria; 10 Department of Nuclear Medicine, Cliniques Universitaires St-Luc and Institute for Experimental and Clinical Research (IREC), Université Catholique de Louvain, Brussels, Belgium; 11 Cap Clinic, Senior Consultant Servei de Radiofísica i Radioprotecció, Hospital de la Santa Creu i Sant Pau, Barcelona, Spain; 12 Canarian Comprehensive Cancer Center, San Roque University Hospital, Las Palmas, Spain; 13 Fernando Pessoa Canarias University, Las Palmas, Spain; 14 Applied Radiation Therapy Trinity, Discipline of Radiation Therapy, Trinity College Dublin, Dublin, Ireland; 15 Trinity St. James’s Cancer Institute, Dublin, Ireland; 16 Polytechnic University of Coimbra, IPC-ESTESC—Coimbra Health School, Medical Imaging and Radiotherapy, Coimbra, Portugal; 17 H&TRC—Centro de Investigação em Saúde e Tecnologia, Lisbon, Portugal; 18 Budapesti Műszaki és Gazdaságtudományi Egyetem (BME), Műegyetem rkp. 3, 1111 Budapest, Hungary; 19 National Institute of Oncology, Ráth Gy. U. 7-9, 1122 Budapest, Hungary; 20 Department of Health Sciences, European University, Nicosia, Cyprus; 21 Medical Physics Expert, Hospital Clínico San Carlos, Universidad Complutense de Madrid, Madrid, Spain; 22 University of Malta, Department of Radiography and EFRS representative, Msida, Malta

**Keywords:** Radiology, Workforce, Radiation protection, Education and training, Basic Safety Standards Directive

## Abstract

**Abstract:**

The European Union Radiation, Education, Staffing & Training (EU-REST) study was a European Commission-funded, 24-month project that analysed workforce availability, education and training needs to ensure quality and safety aspects of medical applications involving ionising radiation in the EU and developed staffing and education/training guidelines for key professional groups involved in ensuring radiation safety and quality of medical radiation applications in the EU Member States. This article outlines the origin, development, goals and overall structure of the project.

**Critical relevance statement:**

This article provides a concise overview of the EU-REST project, which analysed the workforce availability of health professionals involved in the use of ionising radiation for diagnostic and therapeutic procedures and the corresponding education and training in radiation protection.

**Key Points:**

- The aims, professional groups, components, and findings of The European Union Radiation, Education, Staffing & Training (EU-REST) study are described.
- The limited amount of data and literature on staffing recommendations constituted an important finding of the project.
- One of the study’s recommendations is for each EU Member State to maintain a central registry of professionals involved in ionising radiation as well as on related equipment.

## Introduction

In spring 2022, a call for tenders was issued by the European Health and Digital Executive Agency (HaDEA), acting under a mandate from the European Commission’s Directorate General for Health and Food Safety (DG SANTE), in collaboration with the Directorate General for Energy (DG ENER), for a project to identify the current status of workforce availability, education and staffing in medical applications involving ionising radiation within the European Union (EU), and to issue guidelines for appropriate standards in these areas. The project was funded by the EU4Health Programme of the EU [1]; it forms part of the actions of the Strategic Agenda for Medical Ionising Radiation Applications (SAMIRA) Action Plan and contributes to the implementation of Europe’s Beating Cancer Plan [2].

A contract (service contract HADEA/2022/OP/0003) for this project was awarded to a consortium led by the European Society of Radiology (ESR), comprising the ESR, the European Federation of Organisations for Medical Physics (EFOMP), the European Federation of Radiographer Societies (EFRS) and the European Society for Radiotherapy and Oncology (ESTRO), with input also from other stakeholders, including the European Association of Nuclear Medicine (EANM). The project ran from September 1, 2022 until August 31, 2024, and included a number of work packages and deliverables, culminating in the submission to HaDEA of guidelines and recommendations, and a final publishable report, which has now been accepted by HaDEA, DG SANTE, and DG ENER [3].

The aims of the project were to achieve the following:

- Collect and analyse data on workforce availability, education, and training needs to ensure quality and safety aspects of medical applications involving ionising radiation, as well as related stakeholder mapping;
- Draft guidelines for staffing and education/training for medical and other professionals involved in medical radiation applications in EU Member States and related stakeholder consultation;
- Develop conclusions and recommendations on EU workforce availability, education, and training needs for the quality and safety of medical applications involving ionising radiation and related stakeholder consultation.

In the call for tenders issued by HaDEA, it was stated that one aim of the SAMIRA action plan is to “improve workforce availability, education, and training aiming to mitigate the gaps between workforce supply and demand and ensure that all categories of staff involved in radiology, radiotherapy and nuclear medicine receive adequate education, training and continuous professional development in quality and safety issues”. Furthermore, the project was intended to “address the needs for highly qualified workforce and proper forecasts of staff”.

The study covered radiology, radiotherapy, nuclear medicine, and other medical practices utilising ionising radiation, and the main categories of staff under the Council Directive 2013/59/Euratom (Basic Safety Standards Directive, BSSD) [4] definitions of ‘Practitioner’, ‘Medical Physics Expert’, and staff carrying out ‘practical aspects of medical radiological procedures’. The following six professional groups were included: Radiologists, Nuclear Medicine Physicians, Radiation Oncologists (including Clinical Oncologists—depending on local nomenclature), Medical Physicists/Medical Physics Experts, Radiographers, and Radiation Therapists (RTTs—for countries where this group of workers is independent from the category of Radiographers).

The full title of the project was “Workforce availability, education, and training needs to ensure quality and safety of medical applications involving ionising radiation in the EU: Status and recommendations”, referred to in abbreviated form as European Union Radiation, Education, Staffing & Training (EU-REST) [5]. The authors intend to summarise the elements of the EU-REST project, as it applies to radiology and radiography (other publications will report on the project from the perspective of the other professional groups involved), in three publications:

1. An overview of the project
2. The current status across the EU, as determined by data collection and benchmarking
3. Guidelines for education, training, and staffing, with conclusions and recommendations.

The ESR team consisted of three radiologists, a nuclear medicine physician (appointed by the EANM), a radiographer, and two experienced project managers. Other consortium members nominated representatives for their professional groups. Work package leadership and tasks were divided among consortium participants, with representatives of each profession involved in each relevant task and work package.

The project team was supported by an Advisory Board (AB) and a Peer Review Group (PRG). The AB was established to provide views on the methodology and results of the work at each step of the project and consisted of relevant stakeholders, including professions using ionising radiation that were not otherwise represented in the project. It included representatives of the following organisations: ESR EuroSafe Imaging, EANM, ESC (European Society of Cardiology)/EAPCI (European Association of Percutaneous Cardiovascular Interventions), ESNR (European Society of Neuroradiology), CIRSE (Cardiovascular and Interventional Radiological Society of Europe), ECO (European Cancer Organisation), patient representation, IAEA (International Atomic Energy Agency), HERCA (Heads of European Radiation Protection Competent Authorities), UEMS (European Union of Medical Specialists), WHO (World Health Organization) as well as the RPE/RPO/MPE Study [6] and the MARLIN [7] project.

The PRG represented the professional groupings involved in the project (radiology, radiography, radiotherapy, radiation oncology, nuclear medicine, medical physics) with proven expertise in professional and educational matters in the relevant professions, who were not otherwise directly involved in the project. The AB and the PRG received the draft deliverables of each project component with the request to provide comments, which the consortium addressed as appropriate prior to submission of the relevant deliverables to the European Commission.

The project was divided into several components, as summarised below.

### Data collection and analysis

As a first step, a survey (the Pre-Survey) was sent to relevant contacts, seeking to identify the appropriate authorities and professional bodies who would be able to provide relevant information on staffing and education/training for each EU country. Subsequently, a longer, comprehensive survey (the Main Survey) was sent to the contacts indicated in the Pre-Survey, as well as to the relevant EU27 national professional societies, radiation protection authorities and medical associations/chambers, to collect information about the current situation regarding workforce availability, education, and training needs of professionals involved with ionising radiation.

The short Pre-Survey was implemented using the online survey tool SurveyMonkey and circulated by the consortium members to appropriate national contacts. The Pre-Survey was also distributed to members of the SAMIRA Steering Group on Quality and Safety (SGQS) by the European Commission.

The Pre-Survey asked for information and contact details for those bodies which would be expected to be able to provide information on workforce numbers, education and training requirements etc. The professions targeted were Medical Doctors, Radiographers, Radiation Therapists, and Medical Physicists. Responses included at least one from all EU27 countries.

The main survey, also implemented in SurveyMonkey, was divided into four sections related to

- Education and training (including CPD/Continuing Education)
- Workforce availability
- Workforce planning
- Quality and safety.

An abbreviated version of the survey was made available for national radiation protection authorities in the EU27 focusing on the quality and safety elements only.

The survey was distributed

- To the different national organisations and competent authorities from the database established through the Pre-Survey
- To the EU27 national professional societies for Radiology/Nuclear Medicine/Radiotherapy/Radiography/Medical Physics through ESR, EANM, ESTRO, EFRS and EFOMP
- To the EU27 national radiation protection authorities through HERCA
- To the EU27 national medical associations/chambers through UEMS.

Response rates varied depending on the type of professionals or organisations. The vast majority of responders were associated with national professional societies, while a very small response rate was received from national competent authorities.

Data was cleaned with the aim of achieving one response from each source (national authority, national society) to be used in the analysis. More detail will be given in the second part [8] of this 3-article series regarding the conduct, responses and analysis of this survey.

General data about EU Member States’ population, as well as number of hospitals and hospital beds, were added to facilitate data comparison.

### Stakeholder mapping

Twelve stakeholder categories were identified, to be consulted about the draft guidelines for staffing and education/training, and the draft conclusions and recommendations on the EU workforce availability, education and training needs. Any identified stakeholders who were not already included in the data collection described in 1. above were also invited to participate in the Main Survey.

Stakeholder categories included European professional societies, European and international organisations and networks in the relevant fields, patient groups and organisations, relevant industry partners, national professional societies, national medical associations, national competent authorities, academic and research clinical institutions, and any other relevant non-governmental organisations (NGOs) with responsibility for aspects of education, training or workforce determination.

### Identification and analysis of existing guidelines

To inform the guidelines on staffing, a literature review of national, EU, and international staffing guidelines was carried out and considered in the context of current and future practice, including, for example, the impact of new technologies and changing roles brought about by artificial intelligence (AI).

To inform the guidelines on education and training, a literature review on national, EU and international recommendations for education and training was performed.

### Stakeholder consultation

Two rounds of stakeholder consultation formed part of the project.

Initially, stakeholders were surveyed about the usefulness and applicability of the draft guidelines for staffing and education & training. A 39% response rate was achieved. The majority of respondents considered the draft guidelines necessary, useful and realistic. Specific suggestions relating to potential barriers to guideline implementation, and how they may be overcome, were collected.

A second round of stakeholder consultation related to the project conclusions and recommendations, achieving a 40% response rate. Responses indicated a high degree of agreement regarding the proposed recommendations. Again, several specific suggestions to improve the recommendations were received. The consortium considered all comments and addressed them, as appropriate, prior to finalising the guidelines and the project conclusions and recommendations, incorporating several of the stakeholders’ suggestions to improve both the final guidelines and project recommendations.

### Development of guidelines

The staffing and education/training guidelines for key professional groups involved in ensuring radiation safety and quality in medical radiation applications were developed by six author groups, representing the relevant professions within the EU-REST study.

The primary objective of the guidelines that have been created is to delineate the minimum requirements for staffing and education/training across all 27 EU Member States.

The staffing guidelines were developed with the aim to offer methodologies for calculating staffing needs applicable for both current and future practice, with potentially changed or expanded roles of professionals, e.g., brought about by AI, whose concrete impact on work time and tasks cannot yet be reliably predicted. This approach ensures the long-term applicability and relevance of the project outputs. The guidelines consider factors such as the level of available equipment, anticipated workload, and the complexities of the practices undertaken. Regardless of the size or complexity of the institution, an essential methodology for calculating the minimum number of staff required for each profession within each discipline has been established as a baseline. The guidelines are based on the findings of the survey conducted among professional organisations, national societies, government agencies, and regulators, coupled with a comprehensive literature review of existing national, EU, and international staffing guidelines.

The education and training guidelines are based on the current status of education and training according to the survey, as well as the specific education and training requirements of the professions considered in this project. The guidelines aim to propose content to meet the fundamental requirement of a common core of knowledge in radiation safety for all professionals based on the BSSD. In addition, the guidelines define knowledge and requirements specific to each professional group, to ensure optimal and safe practice and take into account the impact of new technologies and techniques, increasing workload, the integration of new treatment approaches, and innovations in current and future practice, including Artificial Intelligence and digital health tools. Training requirements encompass not only radiation protection but also the general training necessary for each profession.

### Benchmarking of workforce availability and training

The aim of this part of the study was to benchmark the data collected through the Main Survey against the EU-REST guidelines, as well as against data identified from the literature review (where available). The limited amount of data obtained from the survey as well as of literature on staffing recommendations restricted the possibilities for benchmarking. On the other hand, identification that such available data was very limited was an important finding in itself, as it led to the study’s recommendation for each EU Member State to maintain a central registry of professionals involved in ionising radiation as well as on related equipment. This would facilitate benchmarking each country’s situation against the EU-REST staffing guidelines and support their adoption where the proposed standards are not yet met.

Data on the duration of specialty training, the number of professionals per 1 million inhabitants, age profile/retirement and other factors as far as available are presented in the EU-REST final project report [3], and are explored in greater detail in the second paper of this 3-article series [8].

### Project conclusions and recommendations

The final report of the EU-REST project contained a summary of all stages of the project, the guidelines developed for workforce staffing, education and training, and all recommendations made by the project consortium, and accepted by the project commissioners (HaDEA). These conclusions, guidelines and recommendations, as they apply to radiologists, will be summarised in the third of our articles outlining this project [9].

## Data Availability

European Commission: European Health and Digital Executive Agency, Analysis on workforce availability, education and training needs for the quality and safety of medical applications involving ionising radiation in the EU—Status and recommendations—Final report, Publications Office of the European Union, 2025, https://data.europa.eu/doi/10.2925/2213975.

## Abbreviations

AB: Advisory Board
BSSD: Basic Safety Standards Directive
DG ENER: Directorate General for Energy
DG SANTE: Directorate General for Health and Food Safety
EANM: European Association of Nuclear Medicine
EC: European Commission
EFOMP: European Federation of Organisations for Medical Physics
EFRS: European Federation of Radiographer Societies
ESR: European Society of Radiology
ESTRO: European Society for Radiotherapy and Oncology
EU: European Union
EU-REST: European Union Radiation, Education, Staffing & Training
HaDEA: European Health and Digital Executive Agency
HERCA: Heads of European Radiation Protection Competent Authorities
MARLIN: Medical Applications of Radiation—Learning from Incidents and Near Misses
MPE: Medical Physics Expert
PRG: Peer-Review Group
RPE: Radiation Protection Expert
RPO: Radiation Protection Officer
RTT: Radiation Therapist
SAMIRA: Strategic Agenda for Medical Ionising Radiation Applications
UEMS: European Union of Medical Specialists
WHO: World Health Organization
